# Polydiacetylenyl β-cyclodextrin based smart vesicles for colorimetric assay of arginine and lysine

**DOI:** 10.1038/srep31115

**Published:** 2016-08-09

**Authors:** Eunae Cho, Hwanhee Kim, Youngjin Choi, Seung R. Paik, Seunho Jung

**Affiliations:** 1 Center for Biotechnology Research in UBITA (CBRU), Institute for Ubiquitous Information Technology and Applications (UBITA), Konkuk University, 120 Neungdong-ro, Gwangjin-gu, Seoul 05029, South Korea; 2 Department of Bioscience and Biotechnology, Microbial Carbohydrate Resource Bank (MCRB), Konkuk University, 120 Neungdong-ro, Gwangjin-gu, Seoul 05029, South Korea; 3 Department of Food Science and Technology, BioChip Research Center,Hoseo University, 20, Hoseo-ro 79beon-gil, Baebang-eup, Asan-si, Chungcheongnam-do, 336-795, Korea; 4 School of Chemical and Biological Engineering, Institute of Chemical Processes, College of Engineering, Seoul National University, Seoul 151 744, South Korea

## Abstract

Selective visualization of arginine and lysine has been explored among 20 amino acids using the hybrid conjugate of β-cyclodextrin (β-CD) and polydiacetylene (PDA). The mono pentacosa-10,12-diynyl aminomethyl group was successfully coupled to either the primary or the secondary face of β-CD, where mono-6-amino-6-deoxy-β-CD or mono-3-amino-3-deoxy-β-CD reacted with the *N*-hydroxysuccinimide ester of 10,12-pentacosadiynoic acid. In this combinatorial system, the cylindrical β-cyclodextrin functions as a channel for the introduction of the cationic amino acids to the artificial membrane. The membrane perturbation and aggregation by the target amino acids could be exclusively visualized as a blue to red color change based on the responsive polydiacetylene domain. These interesting findings demonstrated that the developed β-CD conjugated PDA system may offer a new method of cell-penetrating mechanism, a promising vector system, as well as impact the production industry of arginine or lysine.

Cyclodextrins (CDs) are α-1,4 linked cyclic oligosaccharides consisting of 6 (in α-), 7 (in β-), 8 (in γ-) glucose units. Based on the cylinder-shaped cavities, they possess the molecular recognition and complex-forming ability of various compounds, and thereby make solubility enhancement, chiral separation, and controlled delivery, possible^1^,^2^,^3^. Among these, β-CDs are generally used due to their easy availability and appropriate size, and they have attracted great interest in food, chemical, cosmetic, and agricultural industries^4^. Recently, β-CD functionalized polymers as well as β-CD derivatives have been developed to improve the intrinsic properties and widen the application fields^5^,^6^,^7^. In particular, β-CD-fatty acid conjugates showing amphiphilicity are able to self-assemble to vesicles to encapsulate substances or ingredients.

Diacetylene fatty acids spontaneously arrange into vesicular structures in aqueous conditions, and are polymerized via 1,4-addition under UV irradiation. The resulting polydiacetylene (PDA) has alternating ene-yne structures and shows distinctive electronic and optical properties due to the delocalized pi-conjugation system. Furthermore, the distortion of PDA array by thermal, mechanical or chemical stimuli induces a blue-to-red color transition and an interesting fluorescent response^8^,^9^,^10^. With these structural characteristics, PDA has been utilized as a fascinating sensor platform for interesting target molecules such as antimicrobial peptides, oligonucleotides, and volatile organic acids^11^,^12^,^13^; here, we have designed a novel functionalized PDA with β-CD having inclusion capability, and it is evaluated as smart PDA vesicles showing selective colorimetric response targeting amino acids. In this combinatorial system, β-CD was rationally introduced into a diacetylene monomer to work as a channel for cationic amino acids. The following membrane pertubation and aggregation can be exclusively visualized as a blue to red color change based on the responsive PDA domain (Supplementary Information, Fig. S1).

L-arginine was first isolated from a lupine seedling extract in 1886, and is degraded by arginase to form L-ornithine and urea in mammals, completing ammonia excretion^14^. L-arginine plays a critical role in cell division, wound healing, immune function, and hormone secretion^15^, and the reduced plasma and tissue arginine levels are characterized in septic patients^16^. L-lysine is an essential amino acid that plays an active role in growth and development, collagen formation, calcium absorption, and cholesterol regulation. It is abundant in not plant proteins but animal ones, and has been a vital marker for the nutritional value of food^17^. Both basic amino acids play key roles in protein folding, membrane potential sensing, and the actions of cell-penetrating peptides based on their high pK_a_ values and strong electrostatic interactions^18^,^19^,^20^. They are generally produced via fermentative methods due to its economical and practicable advantages. The production organisms were found to be *Bacillus megaterium*, *Corynebacterium glutamicum*, and *Serratia*, and the control or monitoring of fermentation and the bioreactor processes are necessary for biotechnologists^14^,^21^,^22^. In this regards, the specific targeting and detection of produced amino acids is required. Although the determination of certain amino acids has been reported based on liquid chromatography^23^, ampherometry^24^, and gold nanoparticles^25^, it is still of great interest to develop a rapid, selective, inexpensive and simple handling methods.

Herein, we have designed β-CD conjugated PDA vesicles with dual functionality of molecular recognition and signal visualization for specific amino acids. For this, 2 types of pentacosa-10,12-diynyl amidomethyl β-CD (PCDA β-CD) were synthesized using 2 different amine-functionalized β-CD and *N*-hydroxysuccinimide esters of 10,12-pentacosadiynoic acid. The resultant PCDA β-CD was embedded into the PDA array, and its assembly is utilized as the smart vesicle for arginine and lysine.

## Results and Discussion

### Structural analyses of PCDA β-CDs

**Figure 1:**
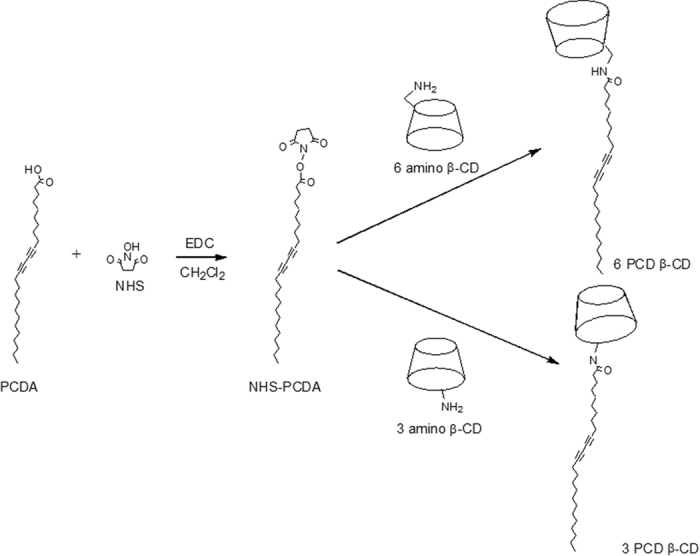
The synthetic scheme of 6 PCD β-CD and 3 PCD β-CD.

**Figure 2:**
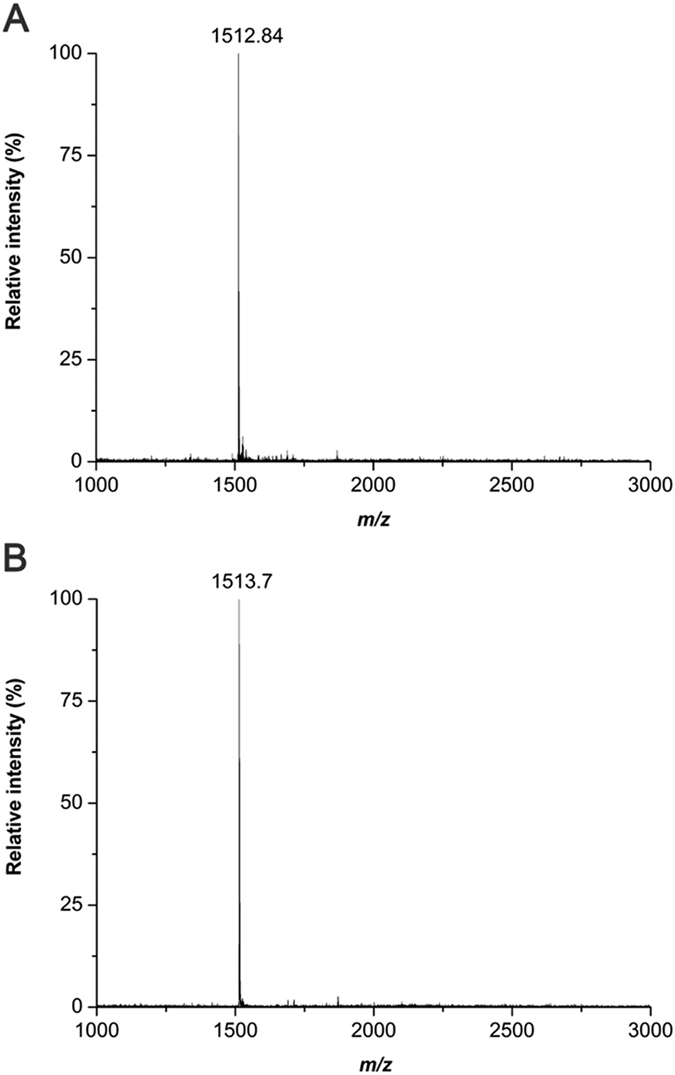
MALDI TOF mass spectra of 6 PCD β-CD (**A**) and 3 PCD β-CD (**B**).

6 PCD β-CD is synthesized by the coupling reaction of 6 amino β-CDs and NHS-PCDA in DMF, as shown in Fig. 1. The 6 amino β-CDs were prepared through amination from 6 tosyl β-CD, the most important intermediate for mono functionalization of β-CD on the primary side. In the same way, PCDA is also functionalized on the secondary side of β-CD for 3 PCD β-CD. The structures are analyzed using MALDI TOF mass spectrometry and NMR spectroscopy. The mass of 6 PCD β-CDs and 3 PCD β-CDs were shown as 1512.84 [6 PCD β-CD + Na]^+^ and 1513.71 [3 PCD β-CD + Na + H]^+^, which were increased in *m/z* 355, due to the substituted mono-pentacosa-10,12-diynyl amidomethyl group (Fig. 2).

**Figure 3:**
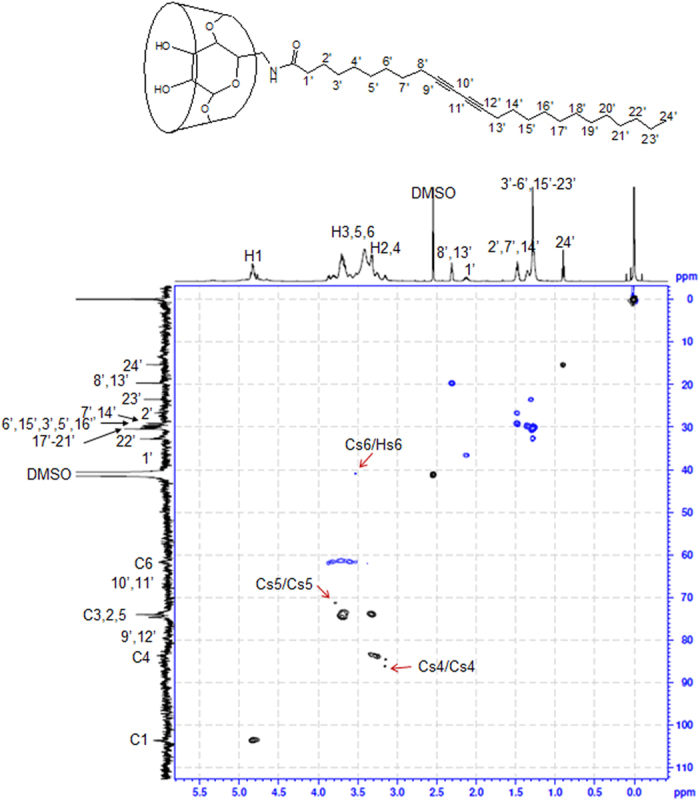
HSQC spectra of 6 PCD β-CD. Solvent: DMSO-*d*_6_. Inset shows the chemical structure.

**Figure 4:**
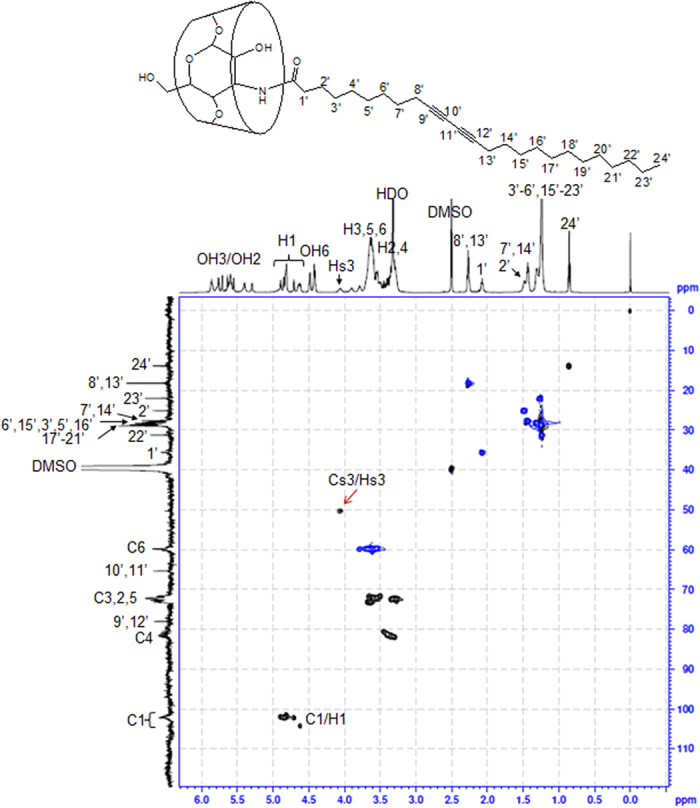
HSQC spectra of 3 PCD β-CD. Solvent: DMSO-*d*_6_. Inset shows the chemical structure.

Figures 3 and 4 show the edited HSQC spectra providing multiplicity information similar to that of a ^13^C DEPT-135 where CH and CH_3_ signals are phased up and CH_2_ signals are phased down^26^. In the DEPT-HSQC spectra, CH_2_ correlation and other multiplicities (CH and CH_3_) are distinguished in blue and black. In the case of the 6 PCD β-CD, the C_s_6 carbons shifted significantly upfield to 41.17 ppm, compared with C6 carbons at 61.58 ppm, which correlated with the methylene germinal H_s_6 signals attached to the C6 carbons (Fig. 3). A moderate downfield shift for H_s_5 (3.76 ppm) and a moderate upfield shift (3.14 ppm) for H_s_4 were correlated with C_s_5 and C_s_5, respectively. In addition, the correlations around 4.88–4.73 ppm for ^1^H and at 103.50 ppm for ^13^C were assigned. The characteristic difference noted for 3 PCD β-CD was observed by the correlation at 4.10 ppm for H_s_3 and 50.16 ppm for C_s_3 (Fig. 4). The anomeric protons were separated ranging from 4.59 to 4.91 ppm, and were attached to the C1 carbons (101.05–104.40 ppm), differently from the 6 positional substitutions of β-CD. Other peaks were all designated, and these structural analyses indicate that 2 types of PCDA β-CDs were successfully synthesized.

### Colorimetric response of β-CD conjugated PDA to amino acids

**Figure 5:**
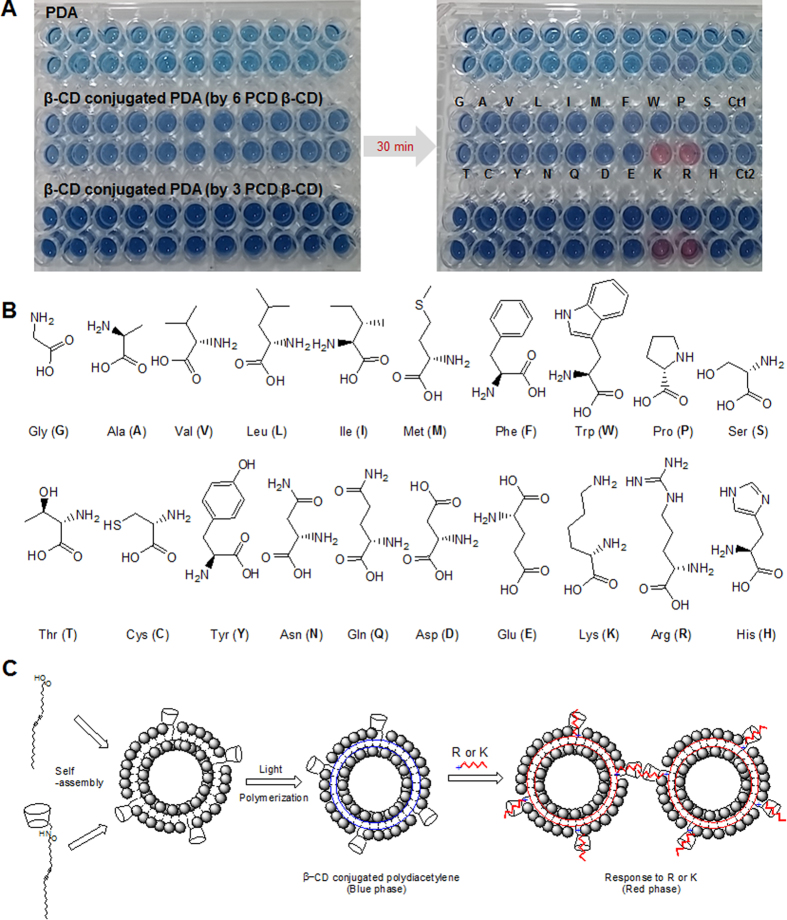
(**A**) Optical images of original PDA and β-CD conjugated PDAs at room temperature after the addition of 20 amino acids. Each cell contained 96 μL of 1 mM PDA or 6 PCD β-CD (3 PCD β-CD): PDA (1:9 mole ratio). The total amino acid concentration was 4 mM. Ct1: no amino acid and HEPES buffer (4 μL); Ct2 : no amino acid and DMSO (4 μL). (**B**) The chemical structure of the 20 amino acids used. (C) Co-assembly of PCDA with β-CD to form β-CD functionalized PDA and the schematic illustration of β-CD conjugated PDA for arginine- and lysine-response.

The synthesized 6 PCD β-CD or 3 PCD β-CD were mixed with PCDA (1:9 mole ratio, 10% portion), and self-assembled into vesicles through heating and sonication as described in Methods section. After polymerization via UV irradiation, the intense blue color was observed due to the alternating conjugated triple bond/double bond PDA. Figure 5A shows a portion of a 96-well plate containing original PDA and β-CD conjugated PDA vesicle solutions where identical quantities of amino acids were added separately. The chemical structures of the treated 20 amino acids are also shown in Fig. 5B. The results clearly demonstrate that the addition of lysine and arginine causes the selective blue to red color transition within only the β-CD conjugated PDA vesicle solutions.

The color change of PDA vesicle is related to the integrity of the vesicle. Since the basic amino acids lysine and arginine have high aqueous pK_a_ values of 10.2 and 12.5^27^, polyarginine having a strong electrostatic interaction with negative charged lipids would give rise to the ability to disrupt and penetrate the bio-membrane^28^. In spite of this, simple lysine or arginine have some limitation to be able to perturb the hydrophobic membrane (hydropathy index: −4.5 (arginine); −3.9 (lysine)), and it could be also observed with no response as shown in the original PDA vesicles. Accordingly, various antimicrobial peptides possess amphipathicity consisting of positively charged amino acids and hydrophobic residues for their function^29^,^30^. In the β-CD conjugated PDA vesicles in this study, β-CD might offer a promising path of the cationic amino acids into the membrane surface, facilitating the ionic interactions and hydrogen bonds with the carboxylic head groups. Their interactions can disturb the hydrogen bonds of carboxyl headgroups in PDA^31^, and impose stresses on the PDA backbone. Thus, we suggest that the selective colorimetric response by the addition of arginine or lysine is desired as depicted in Fig. 5C.

### Comparison of 6 PCD β-CD and 3 PCD β-CD

**Figure 6:**
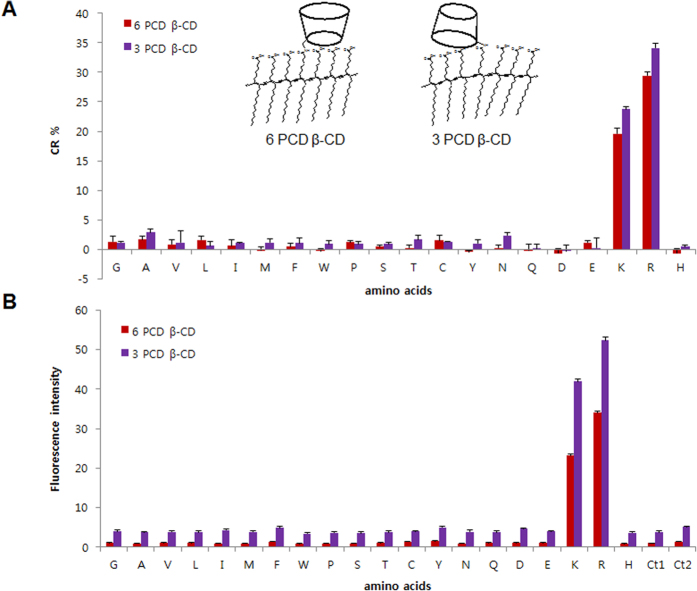
The screening of 20 amino acids for colorimetric response (%) (**A**) and fluorescence intensity (**B**) by β-CD conjugated PDA with 6 PCD β-CD or 3 PCD β-CD. Ct1: no amino acid and HEPES buffer (4 μL); Ct2: no amino acid and DMSO (4 μL). All experiments were performed in triplicate. Insets show the partial states of by β-CD conjugated PDA vesicles with 6 PCD β-CD or 3 PCD β-CD.

To assess the quantitative results of the color change by β-CD conjugated PDA vesicles, the respective CR% was calculated and compared as shown in Fig. 6A. The quantitative assay shows the distinctive values in arginine and lysine, whereas other 18 amino acids do not represent any values distinct from the control: CR% of 6 PCD β-CD = 29% (R), 20% (K); CR% of 3 PCD β-CD = 34% (R), 24% (K). The preferential effect of arginine is attributed to the interaction between guanidium and carboxylate^32^, and the numerical values are higher in 3 PCD β-CD. Although their fluorescence intensities are shown to have a similar pattern and the gradual enhancement was observed as a good linear relationship depending on the concentrations (Fig. 6B and Fig. S4), the relative values are reversed. The 6 PCD β-CD showed the largest fluorescent enhancement in the presence of arginine, and an emission change of up to 33- and 23-fold was observed upon the addition of arginine and lysine (4 mM). In contrast, in the case of 3 PCD β-CD, 14- and 11-fold enhancements were observed in arginine and lysine, respectively.

Since 3 PCD β-CD has the PCDA group directly attached on the secondary side of β-CD, the PDA array was not conserved compared to the original PDA due to the bulky β-CD moiety. In contrast, 6 PCD β-CD has one methylene linker before the substitution of PCDA, and thus the PDA assembly would be more protected than 3 PCD β-CD. Some fluorescent intensities of the other 18 amino acids and controls were also considered due to the little triggered PDA array by the direct grafting of β-CD. Taken together, 6 PCD β-CD is regarded to be more effective for the arginine and lysine-responsive vesicle than 3 PCD β-CD.

### Effect of adamantane carboxylate and the possible mechanism

**Figure 7:**
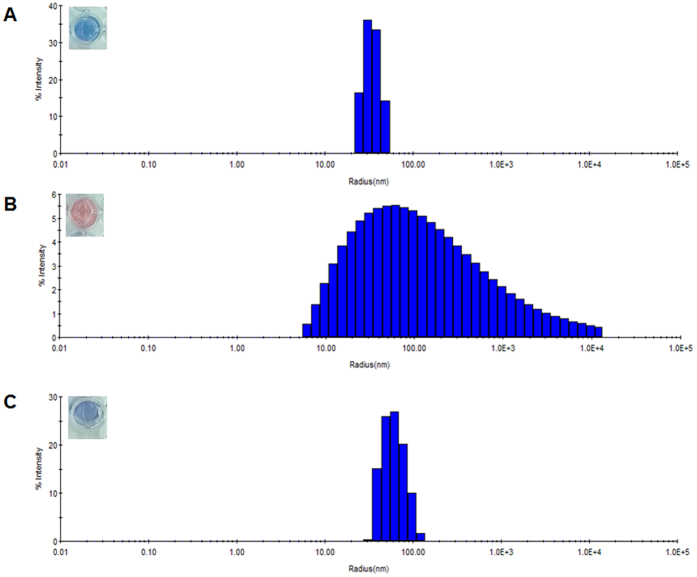
DLS profiles of β-CD conjugated PDA without amino acids (**A**), with 4 mM arginine (**B**), and upon addition of adamantane carboxylate (4 mM) and arginine (4 mM) (**C**). Insets show the corresponding vesicle solutions in 96 well.

The size distribution of β-CD conjugated PDA vesicles by 6 PCD β-CD was investigated using DLS, and the average radius was measured as 33 nm (Fig. 7A). Upon the addition of arginine, the blue to red color transition was observed, and the average size could not be detectable in DLS (Fig. 7B). This result represents aggregation or disruption of β-CD conjugated PDA vesicles in the presence of arginine. Also in protein, the interactions between arginine and carboxylate moieties are the most significant ones occurring between terminal side chains^33^. Furthermore, the microscopic conformational change of polymer chains before and after adding arginine is evidenced by the Raman and fluorescence spectra (Fig. S3). The Raman bands at 1521 and 2111 cm^−1^ corresponding to characteristic C = C and C ≡ C of PDA conjugated backbone are shifted to higher frequency in the presence of arginine. Together with the fluorescence, the result indicates the decrease in conjugation length and planarity of polymer backbone when arginine is loaded onto β-CD conjugated PDA vesicles.

To verify the function of the β-CD cavity, the effect of adamantane carboxylate as the competitive guest was investigated (Fig. 7C). Adamantane carboxylate fits deep in the β-CD cavity, with the carboxylate group exposed to solution at the wider opening of β-CD^34^. Although the size following adamantane insertion was somewhat increased to 63 nm, the arginine could not induce the clear damage on the PDA membrane. The vesicular state was still observed and the blue color was not interrupted. When the cavity is filled with adamantane carboxylate, the entry for arginine into the carboxyl membrane is blocked, and the visible signal is not observed. This result clearly confirms the role of the β-CD cavity in the developed responsive smart vesicle.

**Figure 8:**
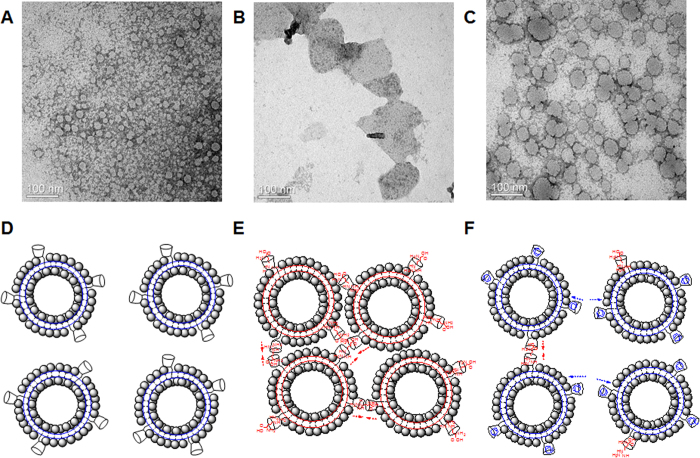
TEM images of β-CD conjugated PDA without amino acids (**A**), with 4 mM arginine (**B**), and upon addition of adamantane carboxylate (4 mM) and arginine (4 mM) (**C**). The plausible states of β-CD conjugated PDA without amino acids (**D**), with arginine (**E**), and upon addition of adamantane carboxylate and arginine (**F**).

As for above 3 samples, TEM image analyses were also conducted. Figure 8A shows the spherical morphology of the β-CD conjugated PDA vesicle, and the general size was found to be about 20 nm. The discrepancy with DLS size may be accounted for by noting that TEM and DLS show solid and swollen vesicles, respectively^35^. When arginine is added to the vesicle solution, the spherical morphology was transformed into the aggregated, assembled and amorphous forms indicating a good agreement with the DLS data and colorimetric response (Figs 7B and 8B). As time goes on, the membrane fusion is accelerated and no specific image is observed. Some cationic polymers have also been reported to induce cell fusion and lateral segregation by the helical conformation and neutralization of the anionic membrane charge^36^. Following the addition of adamantane carboxylate, the spherical morphology was retained, but the larger size and a more concerted state were observed in the vesicle solution (Fig. 8C).

Respective plausible mechanisms are suggested as shown in Fig. 8D–F. Considering 1:1 stoichiometry for arginine: β-CD complex^37^, the arginine might make use of β-CD cavity as a path and attract mutual vesicles (Fig. 8E). The presence of adamantane carboxylate could inhibit the role of arginine by blocking the path (Fig. 8F). Although the guest is captured by the β-CD cavity, the simple binding event cannot affect the pi orbital twist on the PDA array and thereby cannot generate any response of the vesicle^38^. Accordingly, we have designed the channel on the PDA array, and the incorporated cationic amino acids induce the triggering of the ene-yne backbone via a strong electrostatic interactions and hydrogen bonds.

**Figure 9:**
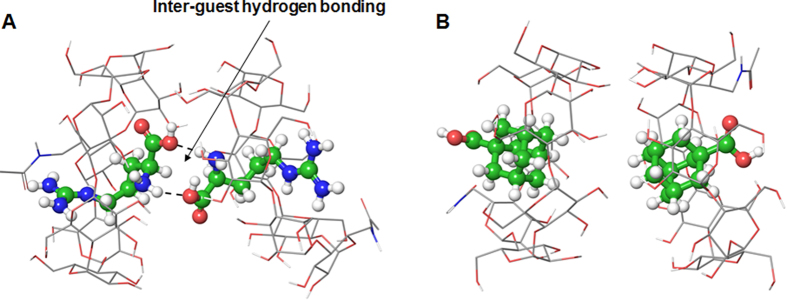
Energy-minimized structures for the (**A**) arginine/6 PCD-β-CD and (**B**) adamantane carboxylate/6 PCD-β-CD complexes. Only arginine/6 PCD-β-CD shows inter-guest hydrogen bonding characteristics by face-to-face positioning of two carboxylic acid groups. The PCDA chains were omitted for image clarity.

To further support the mechanism, we constructed a molecular model of face-to-face interaction for self-assembling of the arginine/6 PCD-β-CD complexes compared with adamantane carboxylate/6 PCD-β-CD. Since bulky PDA chain occupies primary rim space of β-CD, the arginine/6 PCD β-CD complexes are able to interact with each other by their secondary face. This secondary face interaction between 6 PCD-β-CDs can be stabilized by extra hydrogen bonding between guest arginine molecules (Fig. 9A). The energy-minimized complex showed two intermolecular hydrogen bonds between two arginine molecules because carboxylic acid and amino groups are positioned by face-to-face arrangement. However, additional hydrogen bonding stabilization between guest molecules would be impossible for the case of adamantane carboxylate/6 PCD-β-CD complexes (Fig. 9B). The highest-score for the adamantane carboxylate/6 PCD-β-CD docking pose was −4.322 kcal/mol, in which the carboxylic acid group was oriented to primary face of the 6 PCD-β-CD. Because two carboxylic acids are positioned by opposite direction from the secondary face of the 6 PCD-β-CD, inter-guest hydrogen bonding between adamantane carboxylate can be ruled out. This is the molecular basis for the reason of self-assembling feature of arginine/6 PCD-β-CD complex, although they have relatively weak binding score compared with adamantane carboxylate complex.

In summary, in the present study, 2 types of PCDA β-CDs (6 PCD β-CD and 3 PCD β-CD) have been synthesized, and the structures were analyzed using 1D and 2D NMR spectroscopy, and MALDI TOF mass spectrometry. The assembled β-CD conjugated PDAs were evaluated as arginine- and lysine- responsive smart vesicles. Throughout the quantitative assay, DLS, and TEM, the key factors for the functionality of the developed vesicle are suggested to be introducing pores to facilitate the action of specific targets and imposing stresses on PDA array by the dynamic and influential interactions. The present strategy for the selective colorimetric or fluorescent response has the potential to be developed as a signal amplification of PDA sensor system. Furthermore, it would provide a new platform for antimicrobial activity, a promising delivery system as well as a novel method for the production industry of arginine or lysine.

## Methods

### Materials

β-CD, 3-amino-3-deoxy-β-CD and 1-(p-toluenesulfonyl)imidazole were obtained from Tokyo Chemical Industry Co., Ltd. 10,12-Pentacosadiynoic (PCDA), 1-adamantanecarboxylic acid, ammonium chloride, and chloroform were purchased from Sigma-Aldrich Chemicals Co (St. Louis, Mo, USA). *N*-hydroxysuccinimide (NHS) was obtained from Fluka. *N,N*-Dimethylformamide (DMF) was obtained from Alfa Aesar, a Johnson Matthey Company. 1-(3-Dimethylaminopropyl)-3-ethyl carbodiimide hydrochloride (EDC) was purchased from Acros Organics, New Jersey, USA. Organic solvents such as hexane, ethyl acetate, acetone, and diethyl ether were of chromatographic purity, and the water used was triple distilled. L-arginine was purchased from Duchefa Biochemie, Haarlem, The Netherlands. Other amino acids containing L-lysine were obtained from Sigma-Aldrich Chemical Co (St. Louis, Mo, USA) and Tokyo Chemical Industry Co., Ltd.

### Synthesis of mono[6-deoxy-6-(pentacosa-10,12-diynyl amidomethyl)]-β-CD

Mono[6-deoxy-6-(pentacosa-10,12-diynyl amidomethyl)]-β-CD (6 PCD β-CD) was synthesized using 3 steps including amine derivatization of β-CD, NHS activation of PCDA, and their coupling. Firstly, β-CD (5.0 g, 4.4 mmol) was dissolved in water by heating to 60 °C under vigorous stirring. During cooling to room temperature, 1-(p-toluenesulfonyl)imidazole (3.9 g, 17.7 mmol) was added to the suspension. After reaction for 6 h, sodium hydroxide solution was added, and unreacted 1-(p-toluenesulfonyl)imidazole was filtrated. Then, ammonium chloride was added to the filtrated solution to cease the reaction. The resultant mixture was blown by air, and the product began to precipitate out of the solution. After washing, the mono-6-tosyl-6-deoxy-β-CD (6 tosyl β-CD) was dried, and reacted in aqueous ammonia over a 3 day period, refreshing the ammonia solution daily^39^. The obtained mono-6-amino-6-deoxy-β-CD (6 amino β-CD) was confirmed by NMR spectroscopy (Fig. S5).

PCDA (1 g, 2.7 mmoL) was dissolved in 10 mL of dichloromethane. NHS (337.5 mg, 2.9 mmoL) and EDC (615 mg, 3.2 mmoL) were added to the organic solvent^40^. The resulting solution was stirred for 3 h at room temperature. After solvent removal, the product was extracted using ethyl acetate and washed with water. The resulting NHS-PCDA (56 mg, 118.7 μmol) and 6 amino β-CD (100 mg, 88.3 μmol) were reacted in DMF at 45 °C for 24 h. The product was washed, precipitated with ether, and dried overnight (yield: 76.3%). The product was confirmed with an R_f_ value of 0.13 using thin-layer chromatography (TLC, isopropyl alcohol/water/ammonia water 10:3:1). The structure was analyzed using MALDI TOF mass spectrometry and NMR spectroscopy (Figs 2A and 3).

### 6 PCD β-CD

^1^H NMR (600 MHz, DMSO-*d6*): δ 6.60–6.20 (br, O2H, O3H), 4.85–4.80 (m, 7H, H1), 3.88–3.10 (m, 72H, H2–6), 2.31 (t, 4H, H8′, 13′), 2.13 (m, 2H, H1′), 1.48 (m, 6H, H2′, 7′,14′), 1.35–1.28 (br, 26H, H3′–6′ and H15′–23′), 0.90 (t, 3H, H24′); ^13^C NMR (600 MHz, DMSO-*d6*): δ 173.79 (C = ONH), 103.50 (C1), 86.38–82.92 (C4), 79.48 (C9′, 12′), 74.91–70.89 (C3,2,5), 66.72 (C10′, 11′), 61.71 (C6), 36.08 (C1′), 32.70 (C22′), 30.31 (C17′–21′), 29.80–29.59 (C6′, 15′, 3′, 5′, 16′), 29.13 (C7′, 14′), 26.88 (C2′), 23.50 (C23′), 19.71 (C8′, 13′), 15.31 (C24′); MALDI-TOF MS: 1512.84 [6 PCD β-CD + Na]^+^.

### Synthesis of mono[3-deoxy-3-(pentacosa-10,12-diynyl amidomethyl)]-β-CD

The mono-3-amino-3-deoxy-β-CD (3 amino β-CD, 150 mg, 132.4 μmol) is dissolved in DMF (4.7 mL). NHS-PCDA (84 mg, 178.1 μmol) was added to the solution, and reacted at 45 °C for 24 h. After removing the solvent, the residual mixture was washed with diethyl ether and dried in vacuo (yield: 41.6%). The resultant mono[3-deoxy-3-(pentacosa-10,12-diynyl amidomethyl)]-β-CD (3 PCD β-CD) was analyzed using TLC (R_f_ value of 0.23, isopropyl alcohol/water/ammonia water 10:3:1), MALDI TOF mass spectrometry and NMR spectroscopy (Figs 2B and 4).

### 3 PCD β-CD

^1^H NMR (600 MHz, DMSO-*d6*): δ 5.87–5.29 (br, O2H, O3H), 4.89–4.61 (m, 7H, H1), 4.49–4.41 (br, O6H), 4.06 (s, 1H, H_s_3), 3.81–3.21 (br, 56H, H2–6), 2.27 (q, 4H, H8′, 13′), 2.07 (m, 2H, H1′), 1.48 (br, 2H, H2′), 1.43 (m, 4H, H7′, 14′), 1.31–1.24 (br, 26H, H3′–6′ and H15′–23′), 0.86 (t, 3H, H24′); ^13^C NMR (600 MHz, DMSO-*d6*): δ 172.08 (C = ONH), 104.66–100.87 (C1), 82.48–79.89 (C4), 78.00 (C9′, 12′), 73.82–71.00 (C3,2,5), 65.33 (C10′, 11′), 59.77 (C6), 50.30 (C_s_3), 35.60 (C1′), 31.27 (C22′), 29.98 (C17′–21′), 28.67–28.17 (C6′, 15′, 3′, 5′, 16′), 27.88 (C7′, 14′), 25.13 (C2′), 22.06 (C23′), 18.26 (C8′, 13′), 13.91 (C24′); MALDI-TOF MS: 1513.71 [3 PCD β-CD + Na + H]^+^.

### Matrix-assisted desorption/ionization time-of-flight mass spectrometry (MALDI-TOF MS)

Mass spectra were obtained using a MALDI-TOF mass spectrometer (Voyager-DE™ STR BioSpectrometry, PerSeptive Biosystems, Framingham, MA, USA) using the positive-ion mode. 2,5-Dihydroxybenzoic acid (DHB) was used as the matrix.

### Nuclear magnetic resonance (NMR) spectroscopy

A Bruker Avance 600 spectrometer was used to record ^1^H-NMR, ^13^C-NMR, and Distortionless Enhancement by Polarization Transfer-Heteronuclear Single Quantum Coherence (DEPT-HSQC) NMR spectra. All NMR analyses were performed in DMSO-*d*_*6*_ at room temperature.

### Preparation of PDA and β-CD conjugated PDA vesicles

To make original and β-CD conjugated PDA vesicles, PCDA was dissolved in chloroform, and the organic solvent was removed by purging with N_2_ to generate a thin lipid film. Following this, 6 PCD β-CD (or 3 PCD β-CD) and HEPES buffer solution (5 mM, pH 8.0) was added to yield a total concentration of 1 mM. The samples were then heated at 80 °C for 15 min and probe sonicated for 12.5 min using a Sonics VC-505 instrument at 40% of the power used^41^. The resulting solution was filtered through a 0.8 μm filter (Satorius, Minisart), and the milky filtrate was cooled overnight at 4 °C. Polymerization was carried out at room temperature by UV irradiation at 254 nm for 7 min and resulted in a blue color phase.

### Colorimetric response (CR%) measurement

The color transition of the β-CD conjugated PDA vesicles from blue to red was quantified after 30 min, by calculating the colorimetric response (CR%) according to the following equation (1):


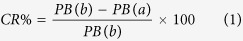


where PB = A_blue_/(A_blue_ + A_red_), A_blue_ is the absorbance at 640 nm for the blue phase, and A_red_ is the absorbance at 550 nm for the red phase. PB(a) and PB(b) are the PB values after and before the addition of amino acids, respectively. The CR% is derived from the change in the ratio of absorbance at 640 and 550 nm in the absence and presence of the specific amino acids.

### Fluorescence detection

Since the blue to red color change of the β-CD conjugated PDA vesicles is accompanied by a generation of fluorescence, the fluorescence intensity was measured after 1 h by a fluorescence microplate reader (Spectramax Gemini EM, Molecular Devices). The excitation wavelength was 485 nm, and the emission was measured at 560 nm.

### Dynamic light scattering (DLS)

DLS measurements were performed using a Wyatt Technology DynaPro Plate Reader to assess the size distribution of vesicles.

### Transmission electron microscopy (TEM)

The vesicles were loaded onto a Formvar-coated copper grid (200 mesh) and air-dried. For negative staining, 2% uranyl acetate solution was used. The nano-vesicles were then examined using energy-filtering TEM (LIBRA 120, Carl Zeiss, Germany).

### Computational Method

Structural characteristics for the 6 PCD-β-CD were calculated using Advanced Conformation Search module in Maestro software (ver. 2016-1, Schrodinger Inc.). Energy-minimized 6 PCD-β-CD structure was traced by conformational searching tool with low-mode sampling method. Enhanced torsional sampling mode was applied with OPLS2005 force-field for maximum 10,000 steps of energy-iterations. Minimum and maximum distance for a low-mode moving was used as 3.0 and 6.0 Å, respectively. The lowest-energy minimum structure for the 6 PCD-β-CD was used as a receptor conformation for the guest arginine during molecular docking simulations.

Glide modeling suite in the Maestro package was used^42^, to conduct docking simulations for the 6 PCD-β-CD and arginine in ligand-flexible mode. The starting structure of arginine was prepared by molecular builder tool in the Maestro software. Docking grid was defined for the 6 PCD-β-CD using the Receptor Grid Generation tool in Glide with the cubic box size of 10 Å. To obtain accurate binding mode and affinity data, docking simulation was performed in the extra precision *XP* mode with Glide XP 5.0 scoring function. In this docking mode, 0.5 kcal/mol of energy window and distance-dependent dielectric constant (*ε* = 2) was applied to pose sampling process. To generate initial docked pose of arginine in the cavity of 6 PCD-β-CD, maximum 100,000 poses were kept for the initial phase of docking and 1,000 poses were energy-minimized using expanded sampling mode. The most 10-highest scoring poses were recorded throughout the docking process.

## Additional Information

**How to cite this article**: Cho, E. *et al.* Polydiacetylenyl β-cyclodextrin based smart vesicles for colorimetric assay of arginine and lysine. *Sci. Rep.* **6**, 31115; doi: 10.1038/srep31115 (2016).

## Supplementary Material

Supplementary Information
