# Supplementary material for: Polydiacetylenyl β-cyclodextrin based smart vesicles for colorimetric assay of arginine and lysine
Source: Sci Rep. 2016 Aug 9;6:31115. doi: 10.1038/srep31115 (PMC4977466; doi:10.1038/srep31115)
Supplement: Supplementary Information [file srep31115-s1.pdf]

Supporting Information

Polydiacetylenyl β-cyclodextrin based smart vesicles for colorimetric assay of arginine and lysine

Eunae Cho,[1] *Hwanhee Kim,*[2] *Youngjin Choi,*[3] *Seung R. Paik,*[4] *Seunho Jung*,*[1],[2]

[1]Center for Biotechnology Research in UBITA (CBRU), Institute for Ubiquitous Information Technology and Applications (UBITA), Konkuk University, 120 Neungdong-ro, Gwangjin-gu, Seoul 05029, South Korea

[2]Department of Bioscience and Biotechnology, Microbial Carbohydrate Resource Bank (MCRB), Konkuk University, 120 Neungdong-ro, Gwangjin-gu, Seoul 05029, South Korea

[3]Departments of Food Science and Technology, BioChip Research Center,Hoseo University, 20, Hoseo-ro 79beon-gil, Baebang-eup, Asan-si, Chungcheongnam-do, 336-795, Korea

[4]School of Chemical and Biological Engineering, Institute of Chemical Processes, College of Engineering, Seoul National University, Seoul 151 744, South Korea

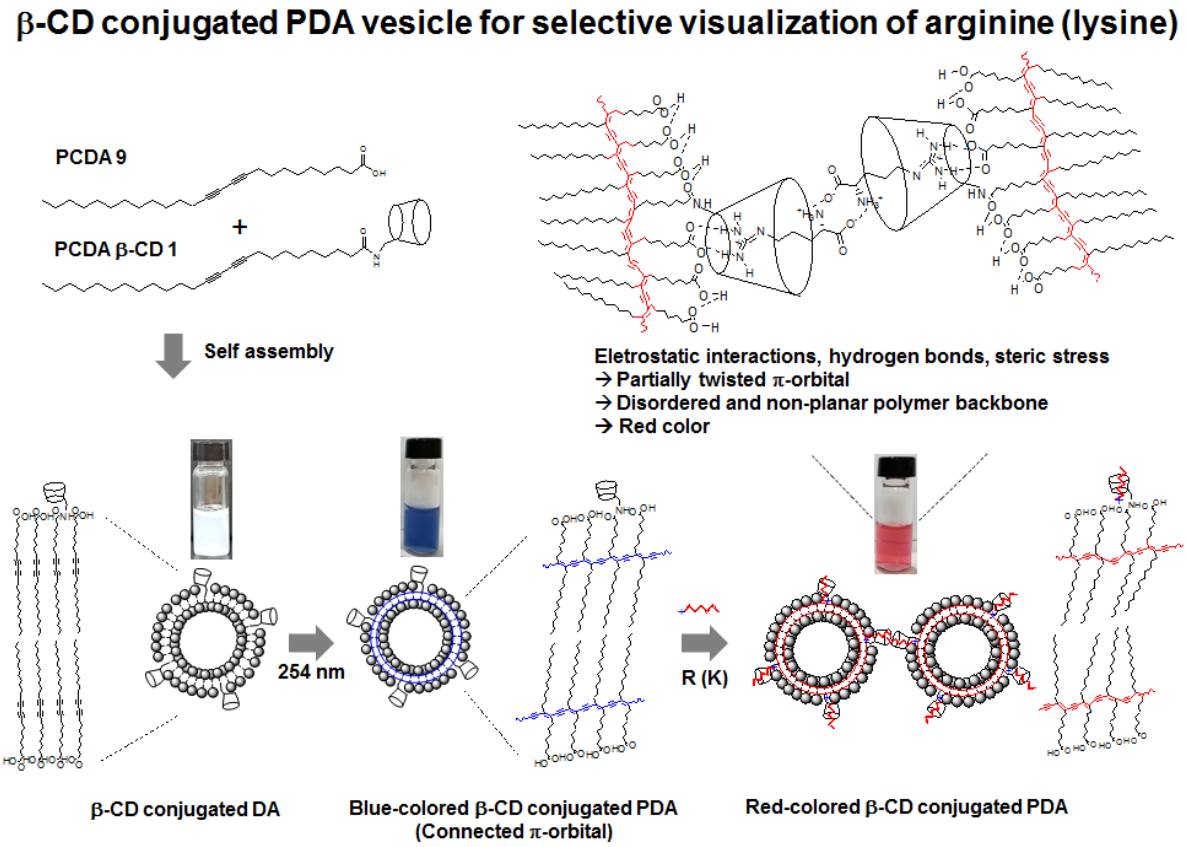

Figure S1. Self-assembly of PCDA β-CD and PCDA to form PDA vesicles and the schematic illustration of PDA vesicles for selective visualization of arginine (lysine).

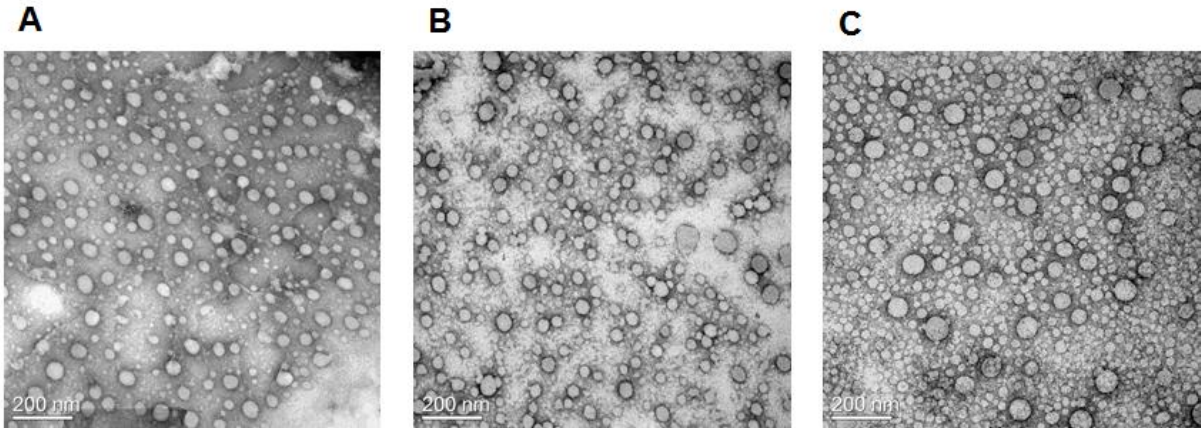

Figure S2. TEM images of original PDA (A) and β-CD conjugated PDAs (10% 6 PCD β-CD (B) and 10% 3 PCD β-CD (C)).

Raman spectroscopy. The vesicle solution was dropped onto a cover glass then dried. The raman spectra were recorded using Horiba Jobin-Yvon T64000 triple stage spectrograph.

Fluoresence spectroscopy. Fluorescence spectra were also taken using a spectrofluorophotometer (RF-5310PC, SHIMADZU). The probe was excited at 485 nm, and the emission spectra were measured in the range of 520-700 nm.

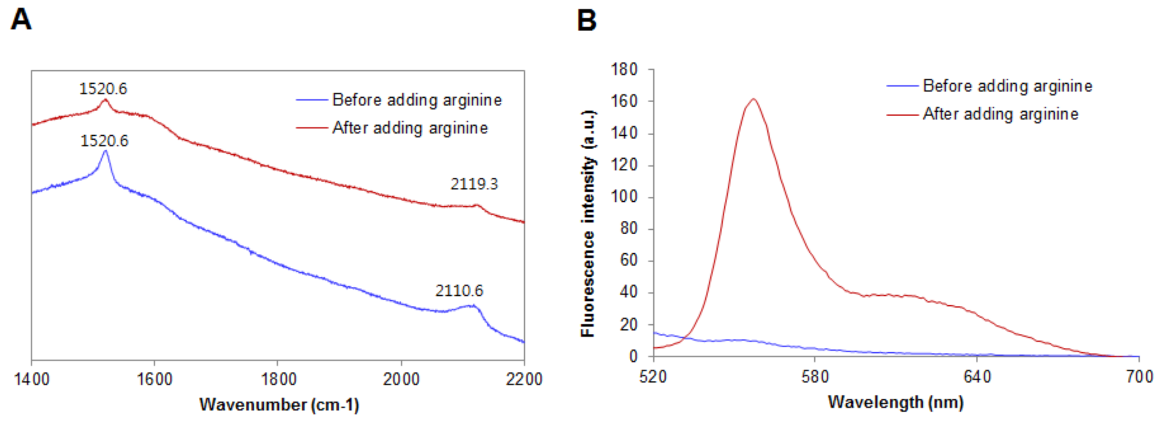

Figure S3. Raman scattering (A) and fluorescence spectra of β-CD conjugated PDAs (10% 6 PCD β-CD) before and after sensing.

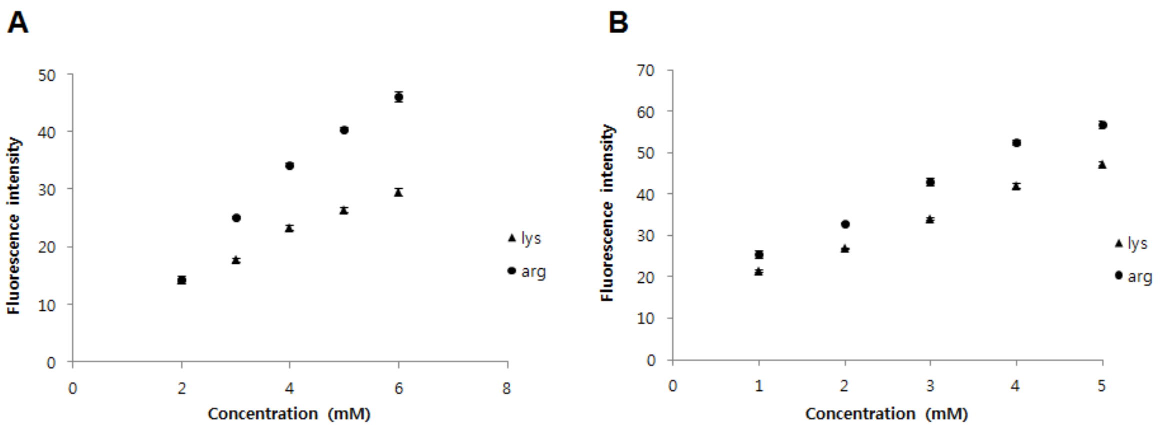

Figure S4. Correlation curve between the fluorescence intensity and the concentration of arginine or lysine in β-CD conjugated PDA with 6 PCD β-CD (A) and 3 PCD β-CD (B). All experiments were performed in triplicate.

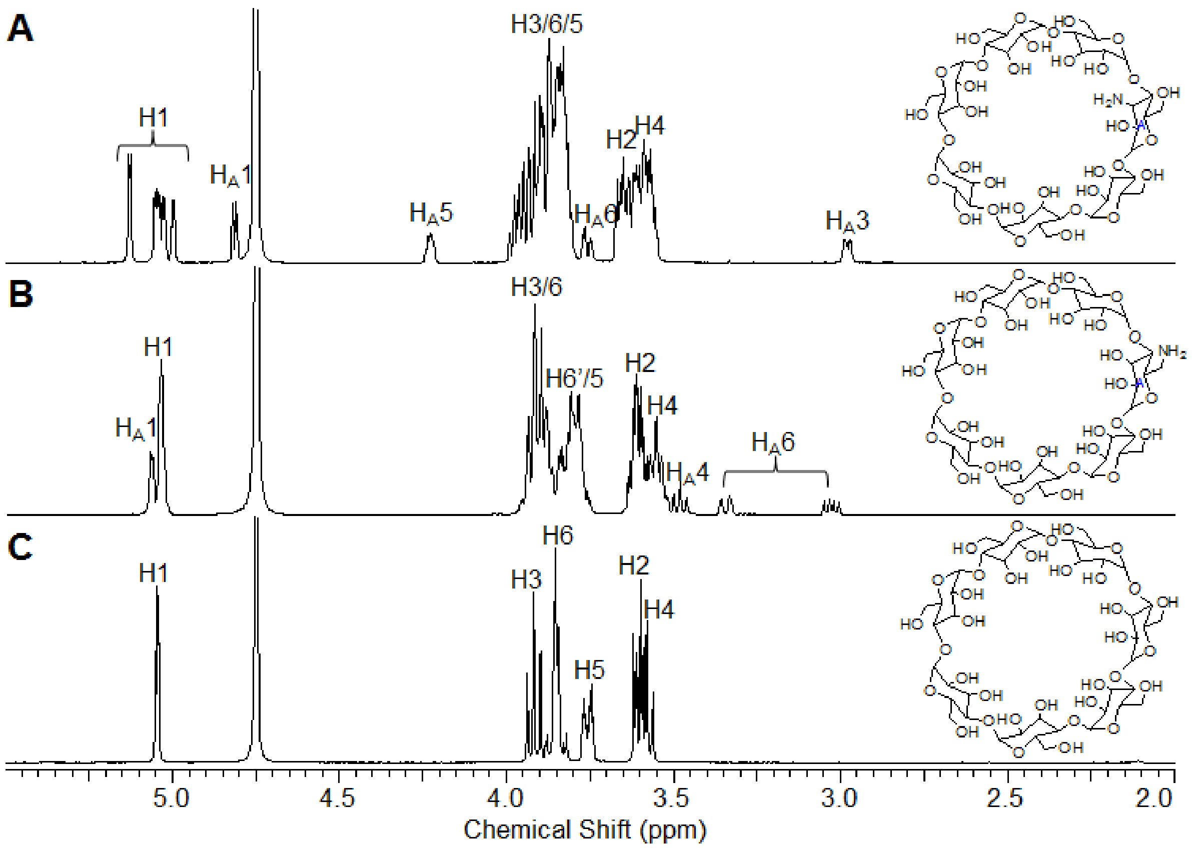

Figure S5. ^{1}H NMR spectra of 3 amino β-CD (A), 6 amino β-CD (B), and unsubstituted β-CD (C). Solvent : D_2O. Inset shows the corresponding chemical structures.

Nuclear magnetic resonance (NMR) spectroscopy. A Bruker Avance 500 spectrometer was used to record NOESY spectra.

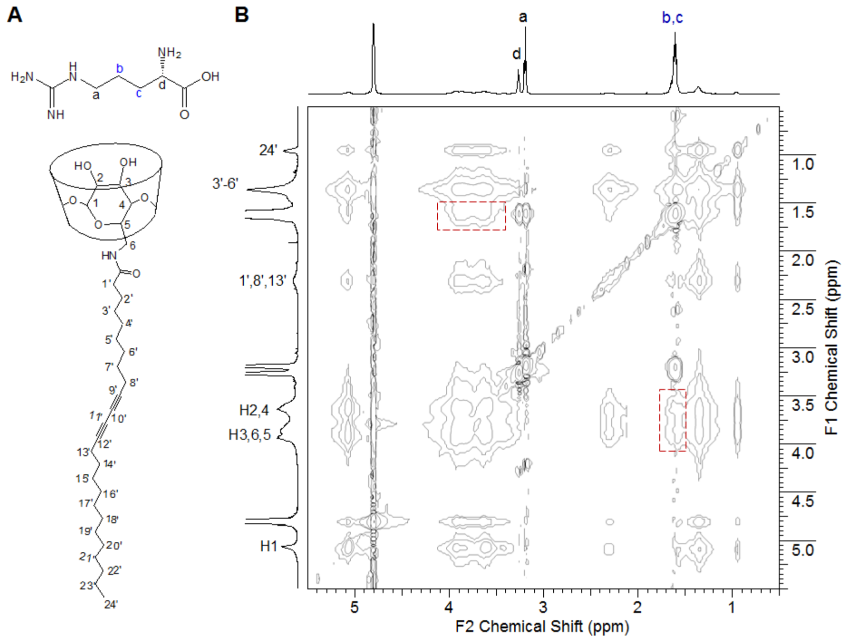

Figure S6. Partial NOESY spectrum of arginine/6 PCD β-CD in D_2O at 298 K with a mixing time of 800 ms. The signals in the F1 and F2 chemical shift are attributed to 6 PCD β-CD and arginine, respectively.

UV-Vis and fluorescence spectroscopy

UV-Vis spectra were obtained with a Shimadzu Corporation UV 2450, UV-Vis spectrophotometry. Fluorescence spectra were also taken using a spectrofluorophotometer (RF-5310PC, SHIMADZU).

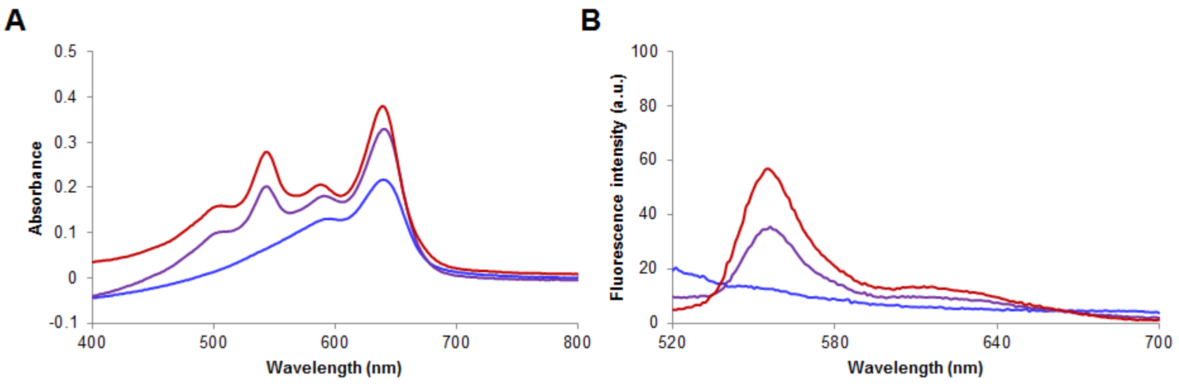

Figure S7. UV-Vis absorption (A) and fluoresence (B) spectra of β-CD conjugated PDA vesicles. (Blue line: 10% 6 PCD β-CD; Purple line: 20% 6 PCD β-CD; Red line : 40% 6 PCD β-CD)

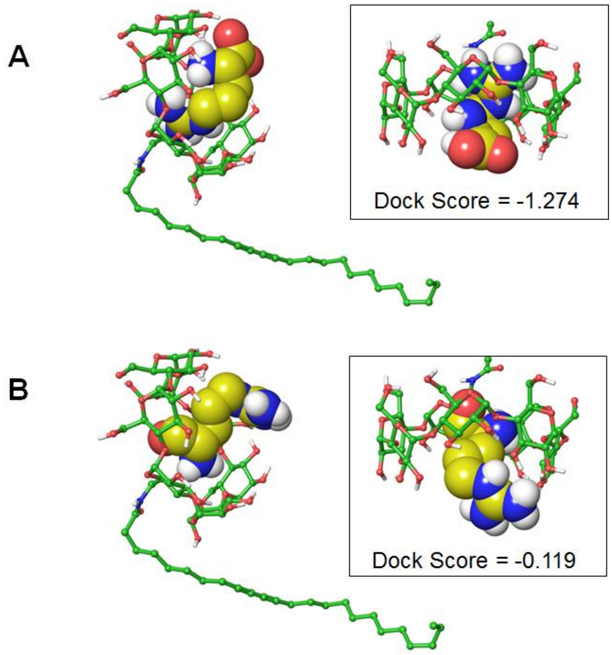

Figure S8. Two different binding modes of inclusion complex for the 6 PCD-β-CD with arginine. (A) guanidium-up, and (B) guanidium-down models were represented by combination of ball-and-stick (6 PCD-β-CD) and space-filling (arginine) rendering.

Docking mode of arginine into 6 PCD-β-CD. For the inclusion complex of 6 PCD-β-CD with arginine, two different binding modes were possible based on side-chain orientation of the arginine; guanidium-up and guanidium-down structures toward primary face of 6 PCD-β-CD. The amino- and carboxylic acid groups of arginine should be oriented to secondary face for the case of guanidium-up structure by definition. For the guanidium-down structure, carboxylic acid group should be oriented to primary face according to same logic. From docking simulation of 6 PCD-β-CD with arginine, the Glide docking score of each guanidium-up and guanidium-down complex was to be -1.274 and -0.119 kcal/mol, respectively. The guanidium-up structure was more stable by 1.155 kcal/mol compared to their downward counterpart. Fig. S8 is snapshot images for the highest-scoring docked pose of both guanidium-up and guanidium-down complex with the 6 PCD-β-CD. Because of its docking score, the guanidium-up and carboxylic acid-down structure of arginine is only considerable to state a theoretical hypothesis on the interaction mode of arginine in the cavity of 6 PCD-β-CD. Therefore the carboxyl moiety should be located on the secondary face of the 6 PCD-β-CD.
